# Conspiratorial Attitude of the General Public in Jordan towards Emerging Virus Infections: A Cross-Sectional Study Amid the 2022 Monkeypox Outbreak

**DOI:** 10.3390/tropicalmed7120411

**Published:** 2022-11-30

**Authors:** Malik Sallam, Huda Eid, Nour Awamleh, Ala’a B. Al-Tammemi, Muna Barakat, Rabaa Y. Athamneh, Souheil Hallit, Harapan Harapan, Azmi Mahafzah

**Affiliations:** 1 Department of Pathology, Microbiology and Forensic Medicine, School of Medicine, The University of Jordan, Amman 11942, Jordan; 2 Department of Clinical Laboratories and Forensic Medicine, Jordan University Hospital, Amman 11942, Jordan; 3 Department of Translational Medicine, Faculty of Medicine, Lund University, 22184 Malmö, Sweden; 4 Department of Dentistry, Queen Alia Military Hospital, Amman 11731, Jordan; 5 Applied Science Research Center, Applied Science Private University, Amman 11931, Jordan; 6 Migration Health Division, International Organization for Migration (IOM), The UN Migration Agency, Amman 11953, Jordan; 7 Department of Clinical Pharmacy and Therapeutics, Faculty of Pharmacy, Applied Science Private University, Amman 11931, Jordan; 8 Department of Medical Laboratory Sciences, Faculty of Allied Medical Sciences, Zarqa University, Zarqa 13132, Jordan; 9 School of Medicine and Medical Sciences, Holy Spirit University of Kaslik, Jounieh P.O. Box 446, Lebanon; 10 Research Department, Psychiatric Hospital of the Cross, Jal Eddib P.O. Box 60096, Lebanon; 11 Medical Research Unit, School of Medicine, Universitas Syiah Kuala, Banda Aceh 23111, Indonesia; 12 Tropical Disease Centre, School of Medicine, Universitas Syiah Kuala, Banda Aceh 23111, Indonesia; 13 Department of Microbiology, School of Medicine, Universitas Syiah Kuala, Banda Aceh 23111, Indonesia; 14 Tsunami and Disaster Mitigation Research Center (TDMRC), Universitas Syiah Kuala, Banda Aceh 23111, Indonesia

**Keywords:** monkeypox virus, orthopoxvirus, social stigma, public health emergency of international concern, misinformation, infodemic, biological warfare, lockdown

## Abstract

Conspiracy theories accompany the emergence of infectious diseases and the 2022 multi-country monkeypox (MPX) outbreak is no exception. It is possible that the adoption of conspiracy beliefs negatively impacts health behavior. We aimed to assess the prevalence of conspiratorial attitudes towards emerging virus infections (EVIs) and the response measures aiming to control these infections among the general public in Jordan. In addition, we assessed MPX knowledge and the belief in the role of men who have sex with men (MSM) in virus spread. The online survey data were collected during 24 May 2022–28 June 2022. The survey instrument was based on previously published scales designed to measure MPX knowledge and EVI conspiracies. A total of 611 respondents formed the final study sample, with a mean age of 44 years and a majority of females (*n* = 433, 70.9%). On a scale ranging from −10 to +10, the median MPX knowledge score in the study sample was +3 (interquartile range: +1 to +5). Educational level was a determinant of MPX knowledge in multivariate analysis. More than 50% of the participants agreed at least to some extent with 9 out of 12 of the EVI conspiracy items. Multivariate analysis showed that embracing conspiracy beliefs about EVIs was associated with being female, and agreeing with or having no opinion regarding the role of MSM in MPX spread. The current study revealed the high prevalence of belief in conspiracies surrounding EVIs, and its accompanying intervention measures, among the general public in Jordan. In addition, a lower level of MPX knowledge was observed compared to previous studies among university students and health professionals in the country. We recommend evaluating the impact of the widely prevalent conspiracy beliefs on health aspects in future studies. This aim is particularly relevant in the Middle Eastern countries where embracing specific conspiracy ideas is a common occurrence.

## 1. Introduction

In humans, the emergence and re-emergence of zoonotic infectious diseases via cross-species transmission is a well-described natural phenomenon [1]. Several factors have contributed to the increased frequency of these spillover events, with continuous occurrence of zoonotic outbreaks at an accelerated rate in the XXI century [2,3]. Possible factors driving anthropozoonosis include climate change, deforestation, animal husbandry, and human contact with wildlife [4,5,6,7].

The direct health effects of zoonotic infections are feared due to the high case-fatality rate reported in some diseases (e.g., Ebola and Marburg virus diseases) [8,9,10]. In addition, the global health effects of these zoonoses, as well as their economic, social and psychological negative impacts, are evident [11]. Furthermore, reporting of infectious disease outbreaks has often been accompanied by a state of anxiety, fear and uncertainty with circulation of mis- and dis-information [12,13,14,15]. In turn, the climate of uncertainty associated with the emergence of infectious diseases represents a suitable milieu for conspiracy theories to flourish [16,17,18]. These conspiracy theories can undermine the trust in health institutions and professionals [19,20]. In addition, evidence is accumulating which points to the negative impact of conspiracy theories on health-seeking behavior and public health intervention measures, including vaccination [21,22,23,24,25,26,27]. Conspiratorial beliefs ushered vaccination since its modern inception against smallpox, and continue to cause damage on public health, particularly in the context of vaccination against pertussis, measles, mumps, rubella and poliomyelitis [28,29,30,31]. 

The multi-country monkeypox (MPX) outbreak in 2022 is a clear example of the prompt infodemic emergence accompanying the reporting of a novel infectious disease [32]. Circulation of unsubstantiated rumors and unscientific beliefs immediately ensued after the early reports of MPX in 2022 [33,34,35,36]. Therefore, the study of the prevalence and scope of conspiratorial beliefs towards emerging virus infections—including MPX—is highly valuable [37,38,39]. This can help in designing effective communication strategies which should be guided by a deep understanding of what people believe [40,41].

Monkeypox is a viral zoonosis that has been endemic in West and Central Africa for more than 50 years, with occasional outbreaks in Europe and the U.S. [42,43]. The disease is caused by the monkeypox virus (MPXV) [43]. This DNA virus is classified within the *Orthopoxvirus* genus together with variola virus, the causative agent of smallpox, which was the first and only infectious disease to be declared eradicated from the human population [44]. The classification of both viruses within the same genus implies the presence of shared features between the two viruses [45]. For example, both viruses cause similar cutaneous clinical features [39,43,46]. In addition, the smallpox vaccination is estimated to be 85% effective against MPX as a result of cross-protection [43,47,48].

Monkeypox is a self-limited disease with variable presentations, particularly during the current 2022 outbreak [43,49,50,51,52,53,54]. Therefore, treatment relies on symptomatic care, with a few antivirals initially designed to treat variola, which can be used in the treatment of MPX as well [43,55]. Antibiotics are not used to treat MPX; nevertheless, it can be used to prevent and manage bacterial superinfection [56]. At present, antiviral agents or Vaccinia immune globulin (SPIG) are recommended for use in a few Middle Eastern countries (e.g., Brincidofovir in Saudi Arabia) [57]. In addition, the Centers for Disease Control and Prevention has also identified other antiviral agents such as Tecovirimat and Cidofovir for the treatment of severe cases and among cases with high risk of severe disease [58]. 

The widespread transmission of MPXV resulted in more than 73,000 cases across 109 countries in the year 2022 alone. Thus, the number of confirmed cases recorded amid 2022 MPX outbreak surpassed the cumulative number of confirmed, probable and suspected cases reported over a period of 50 years [42]. The disease burden varies globally, with the highest number of confirmed cases being recorded in the Americas and Europe, while only 63 cases were reported in nine Arabic-speaking countries of the Middle East and North Africa (MENA) as of 4 October 2022, with a single case in Jordan [47]. 

Prior to the 2022 MPX outbreak, human-to-human transmission of MPXV was reported, particularly in relation to household contact or healthcare settings [59,60]. Virus adaptation to humans may have aided in sustained human-to-human transmission [61,62,63]. Currently, sexual transmission of MPXV among MSM dominates but the disease is not restricted to this group, with cases reported in women and children [52,53,64,65,66,67]. Therefore, specific attention should be paid to prevent the association of the disease with MSM to prevent possible stigma and discrimination towards this high-risk group [68,69,70]. Recently, evidence of human-to-dog transmission of MPXV emerged, which highlights the need for further investigation of the MPX dynamics of spread [71,72].

Upon the first encounter of a novel infectious disease, assessment of the baseline level of knowledge among the general population can be beneficial. This is related to previous evidence indicating that inaccurate knowledge can be linked with higher levels of anxiety, worries and potential conspiratorial ideation in response to the perceived threat of novel infectious agents [15,73]. In addition, closing knowledge gaps can be considered a critical element of the response to an MPX outbreak at the community level [74]. Consequently, filling such knowledge gaps can help in building community resilience for a proper response against infectious disease threats [75,76]. 

Our previous work showed the discernible gaps in knowledge towards MPX among healthcare workers and university students in health schools in Jordan and Kuwait [38,39,77]. In this study, the major aim was to assess the prevalence of conspiracy beliefs towards emerging virus infections among the general public in Jordan. In addition, the study objectives included the assessment of MPX knowledge among the general public residing in the Middle Eastern country. Previous studies in the Middle Eastern Arab countries showed a relatively high prevalence of stigmatizing attitudes towards MSM and towards the patients with sexually transmitted infections [78,79,80]. Thus, we aimed to assess the view of the general public in Jordan towards the role of male homosexuals in the MPX spread worldwide and its links to the embracing of conspiracy beliefs, as an initial step for further studies addressing this specific objective. 

## 2. Materials and Methods

### 2.1. Design

The cross-sectional design was based on a self-administered questionnaire distributed online.

The inclusion criteria, as indicated in the introductory section of the survey, were residence in Jordan and age ≥ 18 years.

### 2.2. Setting

The study questionnaire was distributed online during 25 May 2022–28 June 2022. The questionnaire was created using Google Forms. The anonymous survey was distributed in the Arabic language without monetary incentives for participation. Public posts and direct messages were used to promote participation in the survey using the following social media and instant messaging platforms: Facebook, Twitter and WhatsApp.

Study approval was granted by the Scientific Research Committee—School of Medicine—University of Jordan (reference number: 2544/2022/67). 

### 2.3. Sample and Participants

The minimum sample size needed in this study was calculated using Epitools—Epidemiological Calculators, based on an estimated prevalence of 50%, a desired precision of estimate at 4% and the population size in Jordan, which was about 10,400,000 in 2022 [81,82]. Accordingly, the minimum sample size was estimated to be 601 participants.

### 2.4. Survey Instrument

The questionnaire used in the current study was based on the previously published papers addressing MPX knowledge and conspiracy beliefs towards coronavirus disease 2019 (COVID-19) [38,39,77,83]. The questionnaire was divided into five sections as follows: First, there was an introductory section with general information about the study objectives, followed by the e-consent item (Supplementary File S1). Response to all survey items was mandatory for final submission of a complete form.

Second, the following socio-demographic variables were assessed in a separate section: age, sex, the highest educational level attained (high school or less vs. undergraduate degree vs. postgraduate degree), place of residence (the capital Amman vs. outside the capital), and employment status (employed vs. unemployed).

Third, MPX knowledge was assessed using a ten-item section, with “yes”, “no” and “I do not know” as the possible responses. The MPX knowledge items were based on a previous study by Harapan et al. in Indonesia, with a slight modification as follows [83]: (1) three items evaluating knowledge of the geographic distribution of MPX cases (in the Middle East, West and Central Africa and during the current outbreak); (2) four items assessing knowledge of the viral etiology of the disease, its cutaneous manifestations (skin rash, pustules) and the similarity with smallpox; and (3) three items assessing knowledge regarding ease of transmission, use of antibiotics in treatment and availability of vaccination to prevent the disease.

Fourth, a single item assessed the view of the study respondents towards the role of male homosexuals in the spread of the current MPX outbreak, measured on a 7-point Likert scale ranging from strongly agree through neutral/no opinion to strongly disagree.

Finally, a twelve-item section followed, which comprised the emerging virus infections conspiracy scale (EVICS), based on a previous study by Freeman et al. [37]. This scale was used in our previous work among university students and healthcare workers in Jordan and Kuwait [38,39,77]. The scale items are presented in Table 1.

Each item was measured on a 7-point Likert scale ranging from strongly disagree (+1), to disagree (+2), somewhat disagree (+3), neutral/no opinion (+4), somewhat agree (+5), agree (+6) and strongly agree (+7). Thus, the EVICS possible range was 12–84, with higher scores indicating a higher prevalence of endorsing conspiratorial beliefs regarding emerging virus infections. 

### 2.5. Data Analysis

Data and statistical analysis were conducted using IBM SPSS Statistics for Windows (V22.0. Armonk, NY, USA: IBM Corp).

Level classification for each MPX knowledge item, and for the total MPX K-score, was determined as follows: satisfactory/good knowledge for >70% correct responses; moderate/fair knowledge for 50–70% correct responses; and poor knowledge for <50% correct responses.

Based on the mean EVICS score in the entire study sample, the EVICS score was dichotomized into two categories as follows: EVICS ≥ 56 denoting a higher endorsement of conspiracies regarding emerging virus infections vs. EVICS < 46, indicating a lower level of embracing of these conspiracies.

The Mann—Whitney *U* test (M-W), Kruskal—Wallis test (K-W), chi-squared test and logistic regression multivariate analyses were used as appropriate. The statistical significance level was considered to be *p* < 0.050.

## 3. Results

### 3.1. Characteristics of the Study Participants

The study sample consisted of 611 respondents. The mean and median age of study respondents was 41 years (interquartile range: 30–50 years), with a majority of females (*n* = 433, 70.9%), those with undergraduate degrees as the highest educational level (*n* = 396, 64.8%), those residing in the capital, Amman (*n* = 335, 54.8%), and employed respondents (*n* = 372, 60.9%). Characteristics of the study sample divided by sex is shown in Table 2.

### 3.2. The Prevalence of Conspiracy Ideas Regarding Emerging Virus Infections in the Study Sample

**Figure 1:**
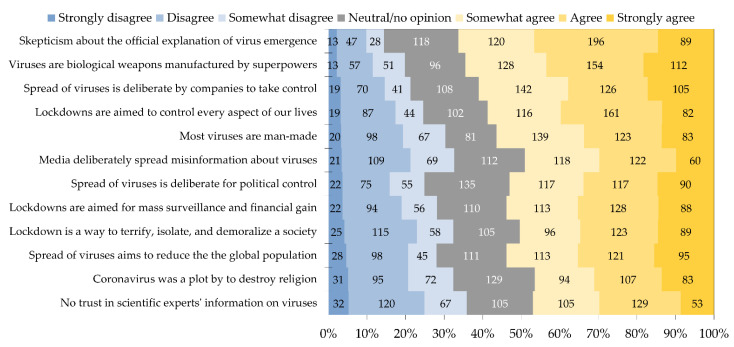
Conspiratorial attitude of the study participants towards emerging virus infections and its subsequent measures.

The overall parameters of EVICS were as follows: mean = 54.8, median = 56.0, standard deviation (SD) = 16.2, interquartile range (IQR): 44.0 to 67.0, and range: 12 to 84. The highest prevalence of agreement with conspiracy items was found for the item assessing skepticism towards the official explanation of virus emergence (*n* = 405, 66.3%), and for the item which assessed the belief of viruses as bioweapons designed by superpowers for global control (*n* = 394, 64.4%, Figure 1).

Univariate analysis showed a stronger belief in conspiracies towards virus emergence among females compared to males (median EVICS: 57.0 vs. 51.0, *p* < 0.001, M-W), while age (*p* = 0.433), educational level (*p* = 0.208), place of residence (*p* = 0.563), and employment status (*p* = 0.842) did not show statistically significant differences.

### 3.3. Monkeypox Knowledge among the General Public Who Participated in the Study

**Figure 2:**
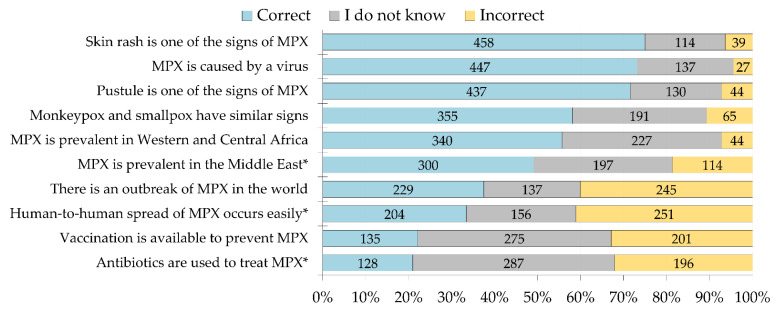
The overall level of monkeypox (MPX) knowledge in the study sample. Knowledge items that are marked with an asterisk represent incorrect statements. * Monkeypox knowledge items that are marked with an asterisk denote incorrect statements.

The overall parameters of MPX K-score were as follows: mean = +3, median = +3, SD = 2.5, IQR: +1 to +5, and range: −4 to +10. Variable levels of MPX knowledge were noticed per item, with the highest percentage of correct responses observed for the items assessing the cutaneous features of MPX, the disease being caused by a virus and the similarity in clinical signs and symptoms with smallpox. The respondents showed satisfactory/good knowledge for the above-mentioned items. A minority of the study respondents knew that antibiotics are not used to treat MPX (*n* = 128, 20.9%), and a minority knew that vaccination is available to prevent the disease (*n* = 135, 22.1%, Figure 2).

Univariate analysis showed a higher MPX K-score among participants with postgraduate degrees compared to undergraduates and those with high school or lower educational attainment (median MPX K-score: 4.0 vs. 3.0 vs. 2.0, *p* < 0.001, K-W), and, among residents in the capital compared to those residing outside Amman (median MPX K-score: 3.0 vs. 2.5, *p* = 0.003, M-W), while age (*p* = 0.747), sex (*p* = 0.136), and employment status (*p* = 0.080) did not show statistically significant differences.

### 3.4. The View of the Study Participants towards the Role of Male Homosexuals in MPX Spread

**Figure 3:**
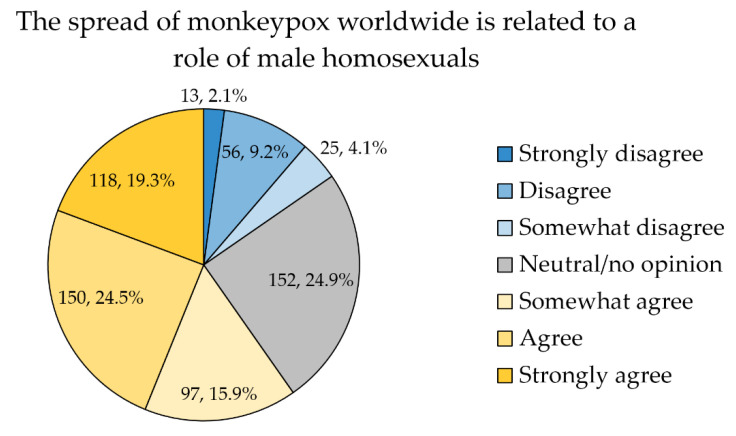
The attitude of the study participants towards the role of male homosexuals in monkeypox spread.

A majority of the study respondents either strongly agreed, agreed to some extent or agreed with the following survey item “the spread of monkeypox worldwide is related to a role of male homosexuals” (*n* = 365, 59.7%, Figure 3). 

Divided into three categories (agreement vs. neutral vs. disagreement), the participants in the agreement category had a higher MPX K-score (mean = 3.2 vs. 2.6 among those in the neutral category vs. 2.6 in the disagreement category, *p* = 0.014, K-W). In addition, the agreement category had a higher mean EVICS compared to the neutral and disagreement categories (58.7 vs. 52.2 vs. 43.8, *p* < 0.001, K-W). Moreover, older age was associated with the agreement category compared to the neutral and disagreement categories (mean: 42 vs. 39 vs. 39 years, *p* = 0.002, K-W). Using the chi-squared test, no significant differences were found based on sex (*p* = 0.401), educational level (*p* = 0.083), place of residence (*p* = 0.337), and employment status (*p* = 0.401).

### 3.5. Conspiracies towards Emerging Virus Infections Were Associated with Female Sex and Agreement That Male Homosexuals Had a Role in MPX Spread

In multivariate analysis, higher EVICS was associated with the female sex (odds ratio (OR) = 1.8) and with the agreement that male homosexuals had a role in MPX spread as opposed to disagreement (OR = 5.2). In addition, the neutral or lack of opinion towards the role of male homosexuals in MPX spread was associated with higher EVICS compared to disagreement (OR = 2.1, Table 3).

## 4. Discussion

In this study, a majority of participants held conspiratorial beliefs towards emerging virus infections. For twelve items that assessed virus emergence conspiracies and conspiratorial ideas towards the subsequent intervention measures, more than 50% of the participants agreed at least to some extent with the content of nine conspiracy items. In addition, the level of agreement exceeded 47% for each of the remaining three conspiracy items. Thus, the results of this study highlighted the high prevalence of specific conspiracy ideas revolving around the explanation of virus emergence and the measures taken to contain it. This observation is consistent with the findings of various studies that were conducted during the COVID-19 pandemic, previous emerging infections including Zika virus and Ebola virus outbreaks and the ongoing MPX outbreak [15,23,37,38,39,73,77,84,85].

Specifically, the pivotal study by Freeman et al. revealed a prevalence ranging between 20% and 50% to endorsing COVID-19 conspiracy beliefs among the general public in the U.K. [37]. Another study from Croatia showed that about 25% of the participants agreed with COVID-19 conspiracy theories [86]. In an early representative survey study involving adults in the U.S., 31% of the participants agreed that severe acute respiratory syndrome coronavirus 2 (SARS-CoV-2) was created and spread on purpose [87]. An earlier study by Piltch-Loeb et al. in the U.S. showed an increase in conspiracy beliefs about Zika fever following domestic transmission of the virus [85]. 

Our previous survey studies that were conducted in Jordan and Kuwait in the context of MPX showed a slightly lower prevalence of these conspiracy ideas compared to the findings of the current study. Using the same survey instrument to assess emerging virus infection conspiracies, the mean EVICS was 43.4 among health students in Jordan, 45.4 among health professionals in Kuwait, and 47.4 among healthcare workers in Jordan [38,39,77]. In this study, the mean EVICS was 54.8, indicating a more conspiratorial attitude towards virus emergence among the general public in Jordan compared to health professionals and university students in health schools in the same country. Taken together, these results show the common occurrence of endorsing conspiracy ideas towards virus emergence in Jordan; nevertheless, the agreement with these ideas was more common among the general public.

A possible explanation for the finding of higher conspiracies among the general public compared to health professionals can be related to higher levels of knowledge about the disease and virus emergence in the latter group. University students in health schools and health professionals are expected to be exposed to topics involving scientific explanations of virus emergence. These topics are covered in courses, workshops and conferences besides other continuous educational activities. In turn, this can reduce the likelihood of embracing conspiracy ideas regarding virus emergence. Nevertheless, previous studies showed the gaps of knowledge regarding emerging infections among healthcare workers, highlighting the need for more focus on the integration of core public health knowledge in medical education [38,39,88,89,90]. Education and improved knowledge could help in addressing conspiracy theories [91]. In spite of this, the current study did not reveal any statistically significant difference in the prevalence of specific conspiracy ideas based on level of education [92,93]. This might highlight the need for a larger sample size to confirm or disprove this observation.

Conspiracy theories can simply be defined as secret plots by powerful entities, and these ideas emerge as an attempt to explain the causes of significant events [94]. Thus, the circulation of conspiracy ideas could be comprehensible amid the emergence of novel or previously neglected infectious diseases [95]. Stemming from the natural need of individuals to understand unexpected events, the embracing of conspiracy ideas becomes evident at times of perceived threat, including the emergence of infectious diseases [95]. Previous evidence indicated that medical conspiracy theories were highly prevalent and can be predictive of several aspects of health behavior [96]. Consequently, conspiracy theories that prevail during infectious disease outbreaks pose significant public health concerns [97]. However, the association between conspiracy theories and negative health behavior remains tentative, taking into account the reporting of an opposite effect by a recent study from Korea [98]. The study by Wang and Kim reported increased preventive actions and vaccination intentions in the context of COVID-19 that were associated with COVID-19 conspiracy theories [98]. The authors linked such a paradoxical finding to several possible hypotheses including the strong collectivist culture in Korea, the low prevalence of conspiracy beliefs, the context of the specific COVID-19 conspiracies which could have a positive influence at time of emergency, as well as the potential measurement bias in the study [98]. 

The assessment of the prevalence of conspiratorial ideas can be the first step of an approach needed to challenge the negative impact of conspiracies on different health aspects [97]. It has been shown that the study of specific conspiracy beliefs can be more helpful in the prediction of health behavior and emotions (e.g., anxiety) [99]. Subsequently, the embracing of specific conspiracy ideas can directly affect engagement in health behavior, as opposed to the effect of the general conspiracy ideation [100]. For example, the compliance with restrictions issued by governments, adherence to preventive actions and the willingness to get vaccinated has been shown to be negatively affected by the embracing of COVID-19 conspiracy beliefs in a study from the U.S. [101]. Another study from Finland demonstrated that the endorsing COVID-19 conspiracy beliefs predicted a lower level of support for governmental restrictions during the pandemic [102]. In addition, our early studies showed a negative association between the vaccine conspiracy beliefs and the willingness to get vaccinated against COVID-19, and human papillomavirus and the uptake of influenza vaccine among healthcare workers [23,103,104]. Furthermore, COVID-19 conspiracy beliefs predicted involvement in public gathering and less adherence to social distancing in a study conducted in Germany and Turkey [105].

In the context of the current MPX outbreak, a recent study that was conducted among the general public in Lebanon showed a prevalence of conspiracy beliefs at 59% [106]. In line with the findings of the current study, the educational level was not linked with the extent of endorsing conspiracy beliefs. In this study, the only sociodemographic factor that was associated with a stronger embracing of conspiracies regarding virus emergence was the female sex. Conflicting results currently exist regarding the sex differences towards conspiratorial thinking. For example, in the Lebanese study by Youssef et al., males were found to embrace conspiracies at a significantly higher level than female participants [106]. On the other hand, our previous work on COVID-19 conspiracies among the general public and students in Jordan and Kuwait showed a higher prevalence of these beliefs among females [15,23,73]. Thus, more studies are need to decipher the role of sex as a determinant of specific conspiracy belief endorsement, particularly in the context of infectious disease and its subsequent effect on health behavior [107].

Despite the relatively high prevalence of endorsing conspiracy ideas about virus emergence in this study, variability was noticed per item. The highest agreement was noticed for three items with >60% level of agreement. Skepticism towards the official explanation of virus emergence was found among about two-thirds of the study sample and 64% agreed at least to some extent with the idea that viruses are bioweapons designed for the purpose of global control. Variability in the endorsement of conspiracy ideas was also evident in the study by Tonković et al. among the general public of Croatia [86]. The Croatian study reported the highest prevalence of agreement with COVID-19 conspiracy ideas for the following items: “the true number of people infected with coronavirus is hidden from the public” (59%), and “the coronavirus did not originate from animals but was created by scientists in the laboratory” (45%) [86]. Our previous studies amid the 2022 MPX outbreak also showed that the highest agreement level with conspiracy ideas were reported for the same content involving virus manufacturing as a bioweapon and skepticism towards the official explanation of virus emergence. This may reflect a relatively high tendency for mistrust in health institutions, policymakers and governments. Subsequently, this mistrust can be associated with worse self-reported health status, and less compliance with public health guidelines and protocols [108,109]. An important issue to be considered for reinforcement of trust in governments and health institutions is to stress on the relevance of transparency, which can prevent the infiltration of conspiracy ideas into new sub-groups within a population [110]. 

Regarding MPX knowledge in this study, and assuming 70% as the cut-off of satisfactory knowledge per item, the participants showed satisfactory knowledge about the cutaneous features of MPX and its viral etiology. Moderate/fair MPX knowledge was found for two items: similarity between MPX and smallpox, and the endemicity of MPX in Western and Central Africa. For the remaining five knowledge items, the correct responses were identified by <50% of the respondents, indicating poor MPX knowledge in the study sample. Defects in knowledge were particularly profound regarding the availability of vaccination and the use of antibiotics for the treatment of this viral infection, with 22% and 21% correct responses, respectively. The later result is particularly worrying, since self-medication and subsequent high prevalence of antimicrobial resistance are commonplace in Jordan [111,112]. In addition, a poor level of knowledge and awareness towards antibiotic use has been reported in the country [113]. Emphasis on the uselessness of antibiotics in the treatment of viral infections including MPX is important since antibiotic misuse during viral outbreaks has been reported, especially during the COVID-19 pandemic [114,115,116].

In line with our findings, recent studies showed unsatisfactory level of MPX knowledge among the general public in several countries in the Middle East. For example, Alshahrani et al. demonstrated a low level of MPX knowledge in a majority (52%) of the study sample involving the general public in Saudi Arabia [117]. However, the low level of knowledge was linked with believing that MPX was a conspiracy or terrorist attack, whereas our results that did not reveal such an association. Another recent study among the general public in Lebanon demonstrated the poor level of MPX knowledge, with less than a third of the participants having a knowledge level equal to or exceeding 60% [118]. Knowledge gaps regarding MPX were also revealed in a recent study among the general public residing in the Kurdistan region of Iraq, with a demonstration of possible mental health issues, including high prevalence of anxiety in relation to a MPX outbreak [119]. An early, extensive study among the general public in Saudi Arabia showed that slightly more than half of the sample had higher-than-average MPX knowledge, while 60% of the participants were worried about MPX becoming a pandemic [120]. A similar level of MPX-related worries were also reported among healthcare workers in the same country [121].

From a global perspective, a recent study among Italian adults by Gallè et al. revealed a low level of MPX knowledge and its prevention measures [122]. Significant gaps in MPX knowledge were also evident in an early study that was conducted among clinicians in Ohio, U.S. [123]. A below-average level of MPX knowledge was also noted regarding the epidemiologic aspects, treatment and immunization among Malaysian dental students [124].

The current study illustrated a higher level of MPX knowledge based on the educational level attained with the highest level of MPX knowledge among postgraduates. Interestingly, the level of MPX knowledge was not linked to the embracing of conspiracies, in contrast to our previous studies among health professionals and university students in health schools in Jordan [39,77]. A previous study in the context of Ebola outbreak showed that higher knowledge was associated with a lower level of embracing of conspiracy [84]. The lack of statistically significant difference with a higher level of embracing of conspiracies among those with lower MPX knowledge seen in this study might be related to the relatively small sample size. 

Despite the previous evidence of an unsatisfactory level of MPX knowledge among health students and health professionals in Jordan, the current study showed even deeper gaps in knowledge among the general public in the same country. This is understandable if the timing of the survey is taken into account, which was at the beginning of MPX reporting, when the information about the outbreak was sparse. Across the three tested categories in the three contemporaneous projects in relation to survey timing, HCWs displayed the highest level of knowledge, followed by health students and, as expected, the general public had the lowest level of knowledge. Previous and recent studies which were conducted in Indonesia, Italy and several Middle Eastern countries among health professionals in general, and physicians in particular showed that there is room for improving the level of MPX knowledge, which can help improve the outbreak response [38,39,43,83,89]. An unsatisfactory level of knowledge coinciding with the onset of an infectious disease outbreak is not an unforeseen result [125,126,127,128]. This highlights the necessity of prompt education, among other interventional measures, to address this barrier which challenges proper outbreak responses. 

Improving knowledge on an emerging virus infection can potentially have a positive effect, as shown previously by Abramson and Piltch-Loeb, during the Zika virus outbreak in the U.S. [129]. Refining MPX knowledge among health professionals and the general public can also be viewed as a prerequisite for effective public health response, preparedness and community engagement in outbreak settings [130]. The primary preventive approach for MPX is to increase the knowledge of the disease for efficient community engagement [131]. In turn, this can be valuable in various ways, including the prevention of stigma directed towards most-at-risk groups and the alleviation of the possible negative psychological, social and economic burden of the disease.

Two interesting results of this study were linked to the agreement with the role of MSM in MPX spread. A majority of the study participants agreed at least to some extent with this statement. The agreement was linked with older age and higher MPX knowledge. Interestingly, the participants who agreed with the role of MSM in MPX spread had higher odds of embracing virus emergence conspiracy beliefs. Although the disproportionately higher occurrence of MPX among MSM networks was evident during the current outbreak, raising awareness that community spread of the virus can occur as well is of utmost importance, especially with cases being recorded in women and children, including neonates [132,133,134]. This can also prevent attaching a stigma of the disease to a particular group [135]. 

### Study Limitations

The current study should be viewed in light of several limitations that included selection and sampling biases based on the sampling approach, measurement bias based on the need to culturally adapt the scales used to the Arabic language, and the social desirability bias in the context of response to EVICS items. Female predominance should be taken into account, especially based on the finding in this study that a greater number of females embrace conspiracies. In addition, the sample size albeit sufficient was relatively small; therefore, it might not be representative of the entire population residing in Jordan. Future studies are recommended to assess the correlation of conspiracy beliefs towards virus emergence, which is a notable phenomenon in the general public in Jordan, with health behavior including attitude to MPX vaccination. Furthermore, our study was limited by the lack of comprehensive socio-demographic data to elucidate possible factors correlated with embracing of conspiracy beliefs and MPX knowledge. Such data in the context of MPX should include monthly income, exposure to animals or pets, history of prior viral illnesses, and history of chronic illnesses.

## 5. Conclusions

The results of the current study point to the widespread prevalence of conspiracy beliefs in a sample of the general public in Jordan. The proliferation of conspiracy ideas towards emerging virus infections could be detrimental on health seeking behavior, which should be investigated in future studies. Thus, a special focus should be directed towards providing an explanation of virus emergence that is scientifically accurate and simplified, to remove any skepticism towards the explanation of infectious disease outbreaks. In this study, proper knowledge about MPX, which is an important determinant of engaging in the preventive efforts, was unsatisfactory. Thus, information campaigns directed towards the general public are encouraged to motivate the community engagement in preparedness and response to the MPX outbreak. These educational measures should consider the provision of concise, scientific messages involving information about virus emergence, and the treatment and prevention of MPX. The issues of stigma and discrimination towards groups with a high risk of contracting MPX should also be prioritized.

## Figures and Tables

**Table 1:** Items of the emerging virus infections conspiracy scale (EVICS).

Item *
I am skeptical about the official explanation regarding the cause of virus emergence
I do not trust the information about the viruses from scientific experts
Most viruses are man-made
The spread of viruses is a deliberate attempt to reduce the size of the global population
The spread of viruses is a deliberate attempt by governments to gain political control
The spread of viruses is a deliberate attempt by global companies to take control
Lockdowns in response to emerging infection are aimed at mass surveillance and to control every aspect of our lives
Lockdowns in response to emerging infection are aimed at mass surveillance and to destabilize the economy for financial gain
Lockdown is a way to terrify, isolate, and demoralize a society as a whole in order to reshape society to fit specific interests
Viruses are biological weapons manufactured by the superpowers to take global control
Coronavirus was a plot by globalists to destroy religion by banning gatherings
The mainstream media is deliberately feeding us misinformation about the virus and lockdown

* The items were adopted from a previous study by Freeman et al. [37].

**Table 2:** Characteristics of the study sample stratified by sex.

Characteristic	Variable	Sex		*p* Value ^2^
		Male N ^1^ (%)	Female N (%)	
Age	≤41 years	101 (56.7)	206 (47.6)	0.039
	>41 years	77 (43.3)	227 (52.4)	
The highest completed educational level	High school or less	56 (31.5)	76 (17.6)	<0.001
	Undergraduate	100 (56.2)	296 (68.4)	
	Postgraduate	22 (12.4)	61 (14.1)	
Residence	The capital (Amman)	100 (56.2)	235 (54.3)	0.667
	Outside the capital	78 (43.8)	198 (45.7)	
Employment	Employed	140 (78.7)	232 (53.6)	<0.001
	Unemployed	38 (21.3)	201 (46.4)	

^1^ N: Number; ^2^ *p* value: Calculated using the chi squared test.

**Table 3:** Multivariate analysis to assess the embracing of conspiracy beliefs regarding emerging virus infections with different study variables.

Factors Associated with Higher Embracing of Conspiracy Beliefs about Emerging Virus Infections ^1^	Odds Ratio (95% Confidence Interval)	*p* Value
Age: ≤41 years vs. >41 years	0.768 (0.544–1.084)	0.133
Sex: Females vs. males	1.834 (1.239–2.715)	0.002
Highest educational level attained: high school or less vs. postgraduates	1.287 (0.690–2.401)	0.428
Highest educational level attained: undergraduates vs. postgraduates	1.087 (0.651–1.814)	0.749
Residence: the capital (Amman) vs. outside the capital	0.909 (0.645–1.280)	0.585
Employment status: employed vs. unemployed	1.331 (0.916–1.934)	0.134
MPX K-score ^4^: MPX K-score ≤ 3 vs. K-score > 3	1.372 (0.970–1.940)	0.074
Agreement that male homosexuals had a role in MPX ^5^ spread worldwide vs. disagreement	5.332 (3.137–9.063)	<0.001
Neutral opinion vs. disagreement that male homosexuals had a role in MPX spread worldwide	2.065 (1.157–3.686)	0.014

^1^ The emerging virus infections conspiracy scale (EVICS) dichotomized based on the mean EVICS: EVICS ≥ 56 indicates a higher embracing of conspiracies vs. EVICS < 56, which indicates a lower embrace of conspiracies with the former as the reference category. ^4^ MPX K-score: Monkeypox knowledge score; ^5^ MPX: Monkeypox.

## Data Availability

The data used in the current study are available upon request from the corresponding author (M.S.).

## Supplementary Materials

The following are available online at https://www.mdpi.com/article/10.3390/tropicalmed7120411/s1, Supplementary File S1: Consent form and questionnaire translated to English.

## Abbreviations

COVID-19	Coronavirus disease 2019
EVI	Emerging virus infection
EVICS	Emerging virus infections conspiracy scale
IQR	Interquartile range
K-W	Kruskal Wallis test
MPX	Monkeypox
MPXV	Monkeypox virus
MSM	Men who have sex with men
M-W	Mann—Whitney *U* test
OR	Odds ratio
SARS-CoV-2	Severe acute respiratory syndrome coronavirus 2
SD	Standard deviation
