# Supplementary material for: Conspiratorial Attitude of the General Public in Jordan towards Emerging Virus Infections: A Cross-Sectional Study Amid the 2022 Monkeypox Outbreak
Source: Trop Med Infect Dis. 2022 Nov 30;7(12):411. doi: 10.3390/tropicalmed7120411 (PMC9787389; doi:10.3390/tropicalmed7120411)
Supplement: Supplementary file 1 [file tropicalmed-07-00411-s001.zip › tropicalmed-2018529-supplementary.pdf]

Supplementary File S1

Consent form and questionnaire translated to English

Knowledge of human monkeypox and the possible explanations of virus emergence among the general public in Jordan

This project aims to assess the knowledge of the general public in Jordan regarding monkeypox, and their attitude towards possible explanations of virus emergence.

The potential benefits of this research include highlighting the gaps of knowledge regarding the emergent monkeypox disease.

The information provided by you in this questionnaire will be used for research purposes. It will not be used in a manner which would allow identification of your individual responses.

Participation requires being 18 years or older in age and current residence in Jordan.

The survey is estimated to take about 5-10 minutes to be completed.

Principal investigators: Malik Sallam and Azmi Mahafzah

Consent: *

- I agree to participate
- I do not agree to participate (submit form)

Age: *………

Sex: *

- Male
- Female

Educational level * (the highest level of formal education completed);

- Undergraduate
- Postgraduate

Place of residence: *

- Amman
- Outside Amman

Monkeypox knowledge (please answer the following questions based on your current level of knowledge as YES vs No vs. I do not know) *

1. Monkeypox is prevalent in the Middle East:
2. Monkeypox is prevalent in Western and Central Africa:
3. There is an outbreak of human monkeypox in the world:
4. Monkeypox is a viral disease infection:
5. Monkeypox is easily transmitted human-to-human:
6. Monkeypox and smallpox have similar signs and symptoms:
7. Skin rashes on the skin are one of the signs or symptoms of human monkeypox
8. Pustules on the skin are one of the signs or symptoms of human monkeypox:
9. Vaccination is available to prevent human monkeypox:
10. Antibiotics are required in the management of human monkeypox patients:

To what extent do you agree with the following statement? (Strongly agree, agree, somewhat agree, neutral/no opinion, somewhat disagree, disagree, strongly disagree) *

The spread of the current human monkeypox outbreak worldwide is related to the role of male homosexuals.

Attitude towards virus emergence and global/local response to outbreaks/pandemics: (Strongly agree, agree, somewhat agree, neutral/no opinion, somewhat disagree, disagree, strongly disagree) *

1. I am skeptical about the official explanation regarding the cause of virus emergence
2. I do not trust the information about the viruses from scientific experts
3. Most viruses are man-made
4. The spread of viruses is a deliberate attempt to reduce the size of the global population
5. The spread of viruses is a deliberate attempt by governments to gain political control
6. The spread of viruses is a deliberate attempt by global companies to take control
7. Lockdowns in response to emerging infection are aimed for mass surveillance and to control every aspect of our lives
8. Lockdowns in response to emerging infection are aimed for mass surveillance and to destabilize the economy for financial gain

9. Lockdown is a way to terrify, isolate, and demoralize a society as a whole in order to reshape society to fit specific interests
10. Viruses are biological weapons manufactured by the superpowers to take global control
11. Coronavirus was a plot by globalists to destroy religion by banning gatherings
12. The mainstream media is deliberately feeding us misinformation about the virus and lockdown

Thank you for participation

*Mandatory items.
